# Fractionated irradiation of carbon beam and the isoeffect dose on acute reaction of skin

**DOI:** 10.1093/jrr/rrt188

**Published:** 2014-03

**Authors:** Akiko Uzawa, Ryoichi Hirayama, Yoshitaka Matsumoto, Kana Koda, Sachiko Koike, Koichi Ando, Yoshiya Furusawa

**Affiliations:** 1 National Institute of Radiological Sciences, 4-9-1 Anagawa, Inage-ku, Chiba-shi, Chiba 263-8555, Japan; 2 Gunma University, Japan

**Keywords:** skin reaction, high-LET, fractionation, Fe-plot

## Abstract

**Purpose:** The aim of this study was to clear any specific LETs cause change in skin reaction. We irradiated mice feet with mono-energetic and SOBP carbon ions, to obtain dose–response of early skin reaction at different LETs.

**Materials and methods:** *Mice*: C3H/HeMsNrsf female mice aged 4 months old were used for this study. The animals were produced and maintained in specific pathogen-free (SPF) facilities. *Irradiation*: The mice right hind legs received daily fractionated irradiation ranged from single to six fractions. Carbon ions (^12^C^6+^) were accelerated by the HIMAC synchrotron to 290 MeV/u. Irradiation was conducted using horizontal carbon-ion beams with a dose rate of ∼3 Gy/min. We chose the LETs at entrance of plateau (20keV/μm) and the SOBP (proximal: 40 keV/μm, middle: 45 keV/μm, distal: 60 keV/μm, distal-end: 80 keV/μm). The reference beam was ^137^Cs gamma rays with a dose rate of 1.2 Gy/min. *Skin reaction*: Skin reaction of the irradiated legs was scored every other day, between the14th and 35th post-irradiation days. Our scoring scale consisted of seven steps, ranging from 0.5 to 3.5 [ 1]. The skin score analyzed a result by the method that described by Ando *et al.* [ 2]. The Fe-plot proposed by Douglas and Fowler was used as a multifraction linear quadratic model. A plot between the reciprocal of the isoeffect dose and the dose per fraction resulted in a straight line.

**Results:** Required isoeffect total dose increased linearly with the fraction numbers on a semi-logarithmic chart at LET 20–60 keV/µm SOBP beam. The isoeffect total dose decreased with the increase in the LET. However, no increases in isoeffect total dose were observed at few fractionations at 80 keV/µm. (data not shown) Using an Fe-plot, we analyzed the isoeffect total dose to evaluate the dependence on Carbon beam, or gamma ray. When I irradiate it by gamma ray, an Fe-plot shows linearly. But, irradiated by Carbon beam, an Fe-plot bent at low fractions (Fig. 1).

**Conclusion:** The LQ-model-based Fe-plot could not fit skin reaction at few fractions at high-LET.

Clinical Trial Registration number if required: No.

**Fig. 1.**
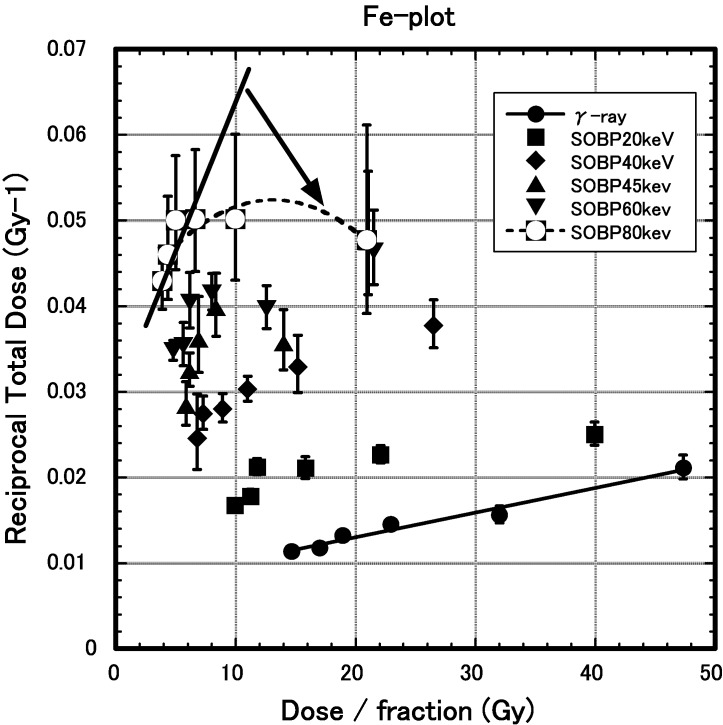
The reciprocal of the isoeffect dose is plotted against the dose per fraction. (i) Gamma ray: Fe-plot was linear. (ii) C-ions: Fe-plot bent at low fractions.

The reciprocal of the isoeffect dose is plotted against the dose per fraction. (i) Gamma ray: Fe-plot was linear. (ii) C-ions: Fe-plot bent at low fractions.
